# Detection of Dengue Virus Serotype 3 Using a Colorimetric Reverse Transcription Loop-Mediated Isothermal Amplification Assay: Evaluation with Clinical Samples from Southeastern Mexico

**DOI:** 10.3390/pathogens15040359

**Published:** 2026-03-28

**Authors:** Perla Pérez-Tepos, Gilma Guadalupe Sánchez-Burgos, Beatriz Xoconostle-Cázares, Gloria María Molina-Salinas, Julio Huchín-Cetz, Edgar Sevilla-Reyes, Berenice Calderón-Pérez, Roberto Ruiz-Medrano, Rosalia Lira

**Affiliations:** 1 Unidad de Investigación Biomédica Oncológica Genómica, Hospital Gineco Pediatría 3A, OOAD Cd Mx Norte, Instituto Mexicano del Seguro Social, Ciudad de Mexico 07760, Mexico; perlav.perezt@cinvestav.mx; 2 Departamento de Biotecnología y Bioingeniería, Centro de Investigación y de Estudios Avanzados del Instituto Politécnico Nacional, Ciudad de Mexico 07360, Mexico; bxoconos@cinvestav.mx (B.X.-C.); bcalderon@cinvestav.mx (B.C.-P.); 3 Unidad de Investigación Médica Yucatán, Instituto Mexicano del Seguro Social, Mérida 97150, Yucatán, Mexico; gilmagburgos@gmail.com (G.G.S.-B.); gloria.molinas@imss.gob.mx (G.M.M.-S.); 4 Laboratorio de Apoyo a la Vigilancia Epidemiológica, Unidad de Investigación Médica Yucatán, Instituto Mexicano del Seguro Social, Mérida 97150, Yucatán, Mexico; juliohc_0203@hotmail.com; 5 Laboratorio de Transcriptómica e Inmunología Molecular, Instituto Nacional de Enfermedades Respiratorias “Ismael Cosío Villegas”, Ciudad de Mexico 14080, Mexico; edgar.sevilla@iner.gob.mx

**Keywords:** dengue virus (DENV), RT-LAMP, colorimetric assay

## Abstract

Dengue virus (DENV), an important mosquito-borne orthoflavivirus, represents a growing global threat due to its geographic expansion and recent outbreaks worldwide. In resource-limited endemic settings, the development of affordable diagnostic assays is needed. In this study, we developed and validated a colorimetric reverse transcription loop-mediated isothermal amplification assay (RT-LAMP) for the detection of DENV type 3 (DENV-3) using 95 previously diagnosed clinical samples from Southeastern Mexico. Primers targeting the 3′ untranslated region (3′ UTR) of DENV-3 were designed, and assay conditions were standardized. The colorimetric RT-LAMP DENV-3 system achieved a preliminary limit of detection of 1 × 10^3^ copies per reaction, with 90.7% sensitivity and 100% specificity. The colorimetric format enabled visual readout without specialized equipment, supporting its potential applicability in point-of-care and resource-limited settings. The developed colorimetric RT-LAMP detection for DENV-3 is intended as a rapid screening/triage tool that can trigger confirmatory testing or public-health actions.

## 1. Introduction

Dengue virus (DENV), an important mosquito-borne orthoflavivirus, represents a growing global threat due to its geographic expansion and recent outbreaks worldwide particularly in low-resource areas [1,2]. In 2023, the region of the Americas reported more than 4.1 million new infections and the number of cases increased threefold in 2024, rising above 13 million reported cases [3]. Notably, DENV serotype 3 (DENV-3), which was previously non-predominant in the region, has been increasingly detected in several countries [4,5]. This shift in serotype prevalence raises concerns regarding the risk of outbreaks and severe clinical outcomes, highlighting the urgent need to strengthen clinical and diagnostic capacities in affected areas [6]. The increase in the circulation of DENV-3 is of particular concern, as population-level immunity may be limited, potentially increasing susceptibility to infection and the risk of severe dengue associated with secondary heterologous infections.

In Mexico, according to data from the Secretariat of Health, a marked increase in DENV-3 cases has been documented in the southeastern region. Confirmed cases rose by 994.4% within a single year (from 5471 cases in 2022 to 54,406 cases in 2023) and by 2287% over a two-year period (reaching 125,160 cases in 2024 compared to 2022). In 2025, there was a considerable decrease in DENV cases, with 21,985 confirmed cases [7,8,9]. These epidemiological trends underscore the critical need for rapid and reliable diagnostic tools that can be deployed in regions experiencing intense viral transmission.

The diagnosis of DENV infection remains challenging in settings where the four antigenically distinct DENV types (DENV-1 to DENV-4) and other arboviruses, like Zika virus (ZIKV) and Chikungunya virus (CHIKV), co-circulate because of the overlapping of clinical presentations. Although molecular methods such as reverse transcription quantitative PCR (RT-qPCR) are considered the gold standard for viral detection and serotyping, their implementation is often limited by infrastructure requirements, cost, and the need for highly trained personnel, especially in point-of-care (POC) settings. In this context, non-PCR-based methods, such as reverse transcription loop-mediated isothermal amplification (RT-LAMP) have been proposed as feasible alternatives for sensitive and specific molecular testing [10]. The LAMP system relies on the strand-displacing activity of *Bst* DNA polymerase and a set of four to six primers that recognize multiple regions of the target sequence, resulting in the exponential amplification of looped DNA structures under isothermal conditions. When coupled with pH-sensitive colorimetric indicators allow visual detection of amplification products without the need for specialized equipment [10,11]. The results can be interpreted by the naked eye, making RT-LAMP particularly attractive for point-of-care deployment [12].

Various RT-LAMP assays for the detection of all four DENV serotypes in clinical samples have been reported, including targeting conserved regions such capsid-premembrane (C-prM) junction [13] and serotype-specific sequences within the 3′ untranslated region (3′ UTR) [13,14,15]. Early and rapid detection of DENV infection during the acute phase is crucial for appropriate clinical management, outbreak control, and public-health responses. Therefore, there is a clear need for accessible, robust molecular diagnostic assays that are easy to perform, require minimal infrastructure, and provide results that can be readily interpreted at the POC.

In this work, we report the development and evaluation of a colorimetric RT-LAMP assay for the detection of DENV-3. The analytical sensitivity and specificity performance of the DENV-3 RT-LAMP colorimetric targeting a conserved 3′untranslated region of the genome were evaluated using serum samples collected from recent outbreaks (2023–2025) in Southeastern Mexico.

## 2. Materials and Methods

### 2.1. Study Design

The aim of this study was to develop a method for detecting DENV-3 RNA in serum samples from individuals undergoing arbovirus diagnostic testing in Mexico. The study protocol was approved by the Ethics Committee (CONBIOETICA-09-CEI-009-20160601) and the Institutional Review Board of the Instituto Mexicano del Seguro Social (R-2016-785-003/R-2026-785-013).

Surplus serum specimens collected for arbovirus diagnosis were obtained from the Epidemiological Surveillance Support Laboratory (Laboratorio de Apoyo a la Vigilancia Epidemiológica/Unidad de Investigación Médica Yucatán; LAVE/UIMY), at IMSS, in Mérida, Yucatán. All samples were anonymized prior to analysis.

The study consisted of an evaluation of the colorimetric RT-LAMP assay for DENV-3 using serum samples collected between 2023 and 2024 from the southeastern Mexican states of Tabasco, Campeche, Yucatán, and Quintana Roo, a region with high dengue incidence. The sample set included DENV-3-positive and -negative sera, as previously determined by RT-qPCR, and corresponding results were retrieved from the laboratory database and used as the reference standard for performance evaluation of the RT-LAMP assay.

### 2.2. Biological Material

#### 2.2.1. Serum Samples

A total of 95 retrospective acute samples collected between 2023 and 2024 were in-cluded, consisting of DENV-positive (n = 65) and DENV-negative (n = 30) sera. Initial testing was performed by the Epidemiological Surveillance Support Laboratory (LAVE/UIMY) IMSS in Mérida, Yucatán, using the TaqMan Triplex Kit (ZIKV/DENV/CHIKV) RT-qPCR (Thermo Fisher Applied Biosystems, Waltham, MA, USA). DENV-positive samples were serotyped using the Dengue Serotyping Real Time PCR Detection Kit (Viasure Certest, Zaragoza, Spain). The samples came from the southeastern region of the country, including the states of Quintana Roo (n = 26 [year 2023 = 16, 2024 = 10]), Yucatán (n = 32 [2023 = 21, 2024 = 11]), Campeche (n = 15 [2023 = 1, 2024 = 14]), and Tabasco (n = 22 [2023 = 9, 2024 = 13]).

#### 2.2.2. Plasmid and Virus Controls

A plasmid-based positive control was generated for DENV-3 RT-LAMP assay. Briefly, the 218 bp fragment corresponding to the region flanked by the F3 and B3 primers within the consensus sequence of the DENV-3 3′ UTR was synthetized by Macrogen Gene Synthesis Service (Macrogen Inc., Seoul, Republic of Korea) in pMG-Amp vector (pMG-DENV3). Plasmid DNA was reconstituted following manufacturer’s instructions and used as DENV-3-positive control. As an endogenous amplification control, primers targeting human 18S rRNA reported by Bartolone et al. [16] were used. For this purpose, a 200 bp fragment of 18S rRNA was amplified by RT-PCR using the external LAMP primers (F3/B3) and a human serum-derived RNA as template, employing the SuperScript™ One-Step RT-PCR System (Invitrogen, Thermo Fisher Scientific, Waltham, MA, USA), following the manufacturer’s recommendations. The amplified product was cloned into the pCR 2.1 vector using the TOPO™ TA Cloning™ kit (Invitrogen, Thermo Fisher Scientific).

For analytical specificity evaluation, RNA serum samples confirmed as positive for DENV types 1, 2 and 3, were provided by the LAVE/UIMY, and DENV-4 RNA was kindly provided by Dra. Martha Yocupicio Monroy from Autonomous University of Mexico City. In addition, ZIKV and CHIKV-RNA were obtained from clarified Vero cell culture supernatants. These viral RNA samples were kindly provided by Dr. Jesús Torres Flores from the Laboratory of Vaccinology and Tropical Diseases from the National School of Biological Sciences, Mexico. The viral RNA was used as cross-reactivity control in the RT-LAMP assays.

### 2.3. DENV-3 RT-LAMP Assay

#### 2.3.1. Primer Design for DENV-3 RT-LAMP Assay

To design primers for the RT-LAMP DENV-3 assay, 425 complete DENV-3 genomes from the Americas were downloaded from the NCBI GenBank database. Sequences were aligned using the MUSCLE algorithm implemented in UGENE software v5.0 (https://ugene.net/ugene/, Unipro), and the alignment was edited to select the 3′ UTR and generate a consensus sequence. Phylogenetic analysis was conducted to assess sequence conservation within the selected region. A neighbor-joining tree was constructed using MEGA X software v11 (https://www.megasoftware.net/) based on the aligned sequences and a logo sequence using the online free software WebLogo (https://weblogo.threeplusone.com/). RT-LAMP primers were designed using the Primer Explorer software v5 (http://primerexplorer.jp/e; Eiken Chemical Co. Ltd., Tokyo, Japan,) following standard LAMP design criteria. The primer set included outer primers (F3 and B3), inner primers (FIP and BIP), and loop primers (LF and LB) to enhance amplification efficiency. Primer sequences are listed in Table 1.

#### 2.3.2. RNA Extraction

RNA was extracted from serum samples using the QIAamp Viral RNA Mini Kit (Qiagen, Hilden, Germany) according to the manufacturer’s instructions. RNA concentration and purity were assessed by spectrophotometry at 260 nm using a NanoDrop 1000 (Thermo Fisher Scientific, Waltham, MA, USA). Extracted RNA was aliquoted and stored at −70 °C until use.

#### 2.3.3. RT-LAMP Assays for DENV-3 and 18S rRNA

The colorimetric RT-LAMP assay for the detection of DENV-3 was performed in a final volume of 6.25 µL, containing 1X of WarmStart^®^ Colorimetric LAMP 2X Master Mix (New England Biolabs, Ipswich, MA, USA), 0.2 µM each of F3 and B3 primers, 1.8 µM each of FIP and BIP primers, 0.4 µM each of LF and LB primers, 1 M betaine (Sigma-Aldrich, St. Louis, MO, USA), and 1 µL of RNA extracted from serum samples or 0.5 ng of pMG-DENV-3-positive control plasmid. For the internal amplification control, a colorimetric RT-LAMP assay targeting human 18S rRNA was performed under same thermal conditions in a final volume of 6.25 µL. Each reaction contained 1X of WarmStart^®^ Colorimetric LAMP 2X Master Mix, 0.2 µM each of F3 and B3 primers, 1.6 µM each of FIP and BIP primers, 0.4 µM each of LF and LB primers, 1 M betaine, and 1 µL of RNA extracted from serum samples or 0.5 ng of pCR2-18S internal control plasmid. Amplification conditions for reactions were 63 °C for 60 min, followed by enzyme inactivation at 80 °C for 5 min, using a T100 thermocycler (Bio-Rad, Hercules, CA, USA). RT-LAMP results were interpreted based on a visible color change from red to yellow, indicative of positive amplification. Samples showing no color change were considered negative. Amplification products were additionally analyzed by 1.4% agarose gel electrophoresis for confirmation, observing the characteristic LAMP ladder-like banding pattern when positive.

#### 2.3.4. Determination of Analytical Sensitivity and Specificity of the DENV-3 RT-LAMP Assay

The copy number (copies/µL) of the plasmid-based DENV-3-positive control was calculated using an online double-stranded (dsDNA) copy-number calculator [17]. Based on these calculations, the plasmid stock concentration was adjusted to 1 × 10^10^ copies/µL. A series of nine 10-fold serial dilutions was subsequently prepared and used to evaluate the analytical sensitivity or limit of detection (LoD) of the RT-LAMP assay. RT-LAMP reactions were performed using each plasmid dilution as template, and all reactions were conducted in duplicate. Assay positivity was defined by a visible color change from red to bright yellow. The LoD was defined as the lowest plasmid concentration consistently producing a positive result in both replicate reactions. Amplification results were further confirmed by agarose gel electrophoresis. Analytical specificity was evaluated using RNA samples confirmed as positive for DENV-1 (Ct 19.2), and DENV-2 (16.37) and DENV-4 RNA was obtained from a reference strain, to assess type specificity. In addition to the plasmid-based DENV-3-positive control, DENV-3 RNA (Ct 16.04) was included as a biological positive control to confirm assay performance using clinically derived material. Cross-reactivity was assessed using ZIKV and CHIKV-RNA. All reactions were conducted in duplicate.

#### 2.3.5. Evaluation of the DENV-3 RT-LAMP Assay Using Clinical Samples

The clinical performance of the DENV-3 RT-LAMP assay was evaluated using the RNA extracted from the panel of 95 retrospective human serum samples characterized and provided by LAVE/UIMY. The DENV-3-positive samples (n = 65) had a RT-qPCR cycle threshold (Ct) values < 32. RNA was tested using the colorimetric DENV-3 RT-LAMP assay under the conditions described above. Each RT-LAMP reaction was performed in duplicate. In parallel, RT-LAMP assay targeting 18S rRNA was performed for all samples as internal amplification control to verify RNA integrity and reaction performance. RT-LAMP results were interpreted based on visual color change and confirmed by agarose gel electrophoresis. The results of samples that had previously tested positive for DENV-3 by RT-qPCR but tested negative for DENV-3 by RT-LAMP were confirmed in a second experiment conducted in duplicate.

#### 2.3.6. Quantification of Color Change in the DENV-3 RT-LAMP Reaction

To quantify color change in the colorimetric assays, we analyzed nine DENV-3-RNA samples collected in 2025 and previously confirmed by the Epidemiological Surveillance Support Laboratory (LAVE/UIMY). These samples corresponded to the same RNA extracts that had already been used for diagnostic confirmation with the gold-standard assay. Because the colorimetric LAMP assay contains phenol red, a shift from red (negative) to yellow (positive) is expected as nucleic acid amplification lowers the pH of the reaction mixture. To quantify the color change, absorbance was measured at 434 and 560 nm using a NanoPhotometer^®^ (Implen, Munich, Germany). The absorbance at 560 nm was subtracted from the absorbance at 434 nm to obtain ΔOD, as previously described [18]. Data was plotted using GraphPad Prism version 8.0 (https://www.graphpad.com/; GraphPad Software, Boston, MA, USA).

### 2.4. Statistical Analysis

Diagnostic sensitivity and specificity of the DENV-3 RT-LAMP assay was compared against LAVE/UIMY RT-qPCR results, used as the reference standard method. Samples were classified as RT-LAMP-positive when both a color shift and the characteristic LAMP ladder-like banding pattern were observed on agarose gels. Samples were classified as RT-LAMP-negative when no color change occurred and no amplification was detected by electrophoresis. True positives (TP) and true negatives (TN) were defined as samples positive or negative by both RT-LAMP and RT-qPCR, respectively. False positives (FP) and false negatives (FN) were defined as discordant results between RT-LAMP and RT-qPCR. Diagnostic performance metrics were calculated using the Diagnostic Test Evaluation Calculator (https://www.medcalc.org; MedCalc, Ostende, Belgium, accessed on 21 November 2025).

## 3. Results

### 3.1. Development and Standardization of the DENV-3 RT-LAMP Assay

**Figure 1:**
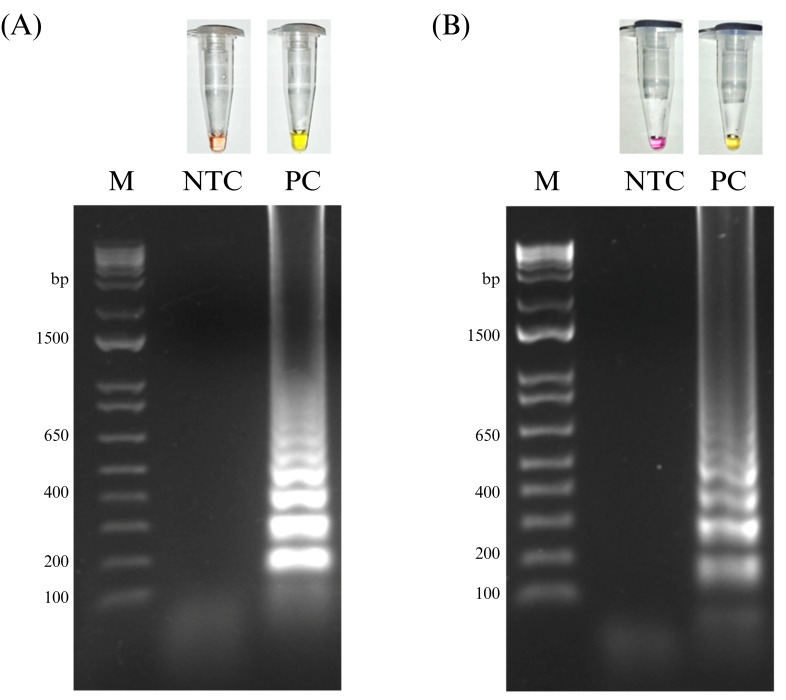
Colorimetric RT-LAMP DENV and 18S rRNA assays. Representative results of the colorimetric RT-LAMP assay for DENV-3 targeting 3′ UTR (**A**) and 18S rRNA as internal amplification control (**B**). Upper panes show colorimetric readout of the RT-LAMP reactions, where a color change from red/pink to yellow indicates positive amplification. Lower panels show agarose gel electrophoresis of the corresponding RT-LAMP products, with positive reactions displaying the characteristic ladder-like banding pattern. M: DNA molecular marker; NTC: non-template control; PC: positive control.

A conserved target region within the 3′ UTR of DENV-3 was identified for RT-LAMP primer design. A 218 bp consensus sequence was generated from a multiple sequence alignment of 425 complete DENV-3 genomes originally from the Americas and available in NCBI GenBank. The dataset included sequences from Bolivia (n = 1), Brazil (n = 88), Colombia (n = 20), Ecuador (n = 2), Guyana (n = 1), Nicaragua (n = 142), Paraguay (n = 6), Peru (n = 8), Venezuela (n = 113), and Mexico (n = 3) (Figure S1). Based on this sequence, a set of six primers (F3, B3, FIP, BIP, LF, and LB) was designed for the DENV-3 RT-LAMP assay (Figure S1). To evaluate primer coverage and sequence conservation, a phylogenetic tree of the target region was constructed. NJ phylogenetic analysis of the target region confirmed high conservation with branch lengths for 99% of sequences < 0.0001 substitutions/site (Figure S2). Alternatively, a logo sequence of the amplification fragment of the DENV-3 RT-LAMP system was performed, showing the high degree of conservation of the region (Figure S3). The DENV-3 RT-LAMP assay was developed using a colorimetric master mix, and reaction conditions yielding consistent and robust amplification were identified and selected for subsequent analytical and clinical evaluation. Under these conditions, the DENV-3 RT-LAMP assay produced a clear colorimetric shift from red to yellow in positive reactions, which was accompanied by the characteristic ladder-like banding pattern upon agarose gel electrophoresis (Figure 1A). In parallel, the RT-LAMP assay targeting human 18S rRNA was evaluated as an endogenous internal amplification control. Serum RNA samples produced consistent positive amplification of 18S rRNA, confirming RNA integrity and reaction performance (Figure 1B).

### 3.2. Analytical Sensitivity and Specificity of the DENV-3 RT-LAMP Assay

**Figure 2:**
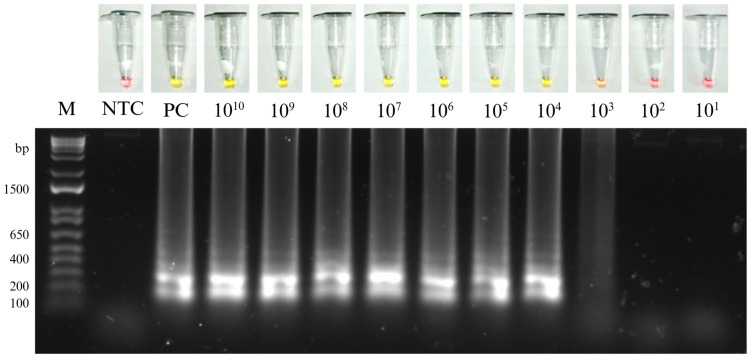
Preliminary analytical sensitivity of the DENV-3 RT-LAMP assay. Colorimetric detection (upper panel) and agarose gel confirmation (lower panel) of the DENV-3 RT-LAMP assay using 10-fold serial dilutions of the pMG-DENV-3-positive control plasmid. The limit of detection was determined based on the lowest plasmid concentration consistently yielding positive amplification. M: DNA molecular marker; NTC: non-template control.

The preliminary analytical sensitivity of the DENV-3 RT-LAMP assay was determined using 10-fold serial dilutions of the plasmid-based DENV-3-positive control (10^10^ to 10^1^ copies/reaction). The DENV-3 RT-LAMP assay reliably detected down to 10^3^ copies/reaction (Figure 2). Reaction containing ≥ 1 × 10^4^ copies produced a clear colorimetric change from red to yellow, whereas reactions containing 1 × 10^3^ copies typically exhibited a reproducible intermediate orange color. This threshold was therefore considered the preliminary limit of detection (LoD) for the assay. Amplification results were corroborated by agarose gel electrophoresis, which showed the characteristic ladder-like banding pattern of LAMP products in positive reactions, while no bands were detected in negative reactions.

**Figure 3:**
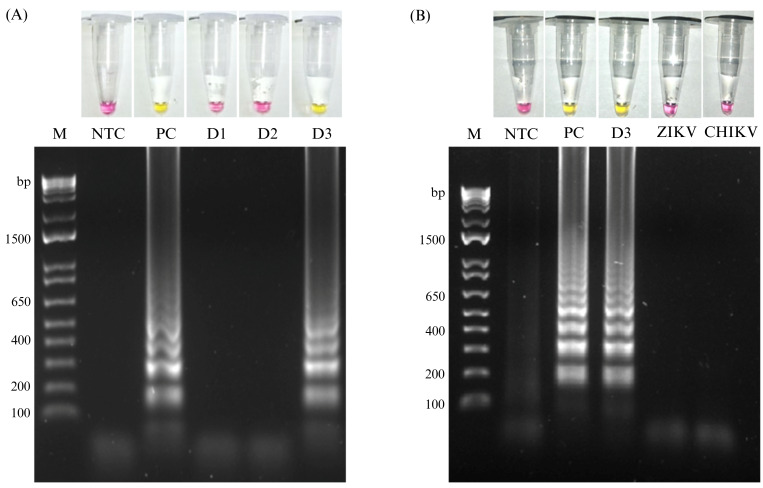
Analytical specificity of DENV-3 RT-LAMP assay. Colorimetric detection (upper panel) and agarose gel confirmation (lower panel). (**A**) Type specificity assessment using RNA samples previously confirmed by RT-qPCR as positive for dengue virus types 1, 2, and 3 (D1, D2, and D3). Only DENV-3 RNA produced a positive RT-LAMP signal. (**B**) Cross-reactivity evaluation using ZIKV and CHIKV-RNA. M: DNA molecular weight marker; NTC: no-template control; PC: positive control.

The analytical specificity of the DENV-3 RT-LAMP assay was evaluated using RNA of DENV-1, DENV-2, DENV-3 and DENV-4. Only the DENV-3–RNA produced a positive LAMP signal, indicating that the assay discriminates DENV-3 from other DENV types tested (Figure 3A). Cross-reactivity was further evaluated using ZIKV and CHIKV-RNA; no color change or amplification by electrophoresis were observed (Figure 3B).

### 3.3. Evaluation of the DENV-3 RT-LAMP Assay with Clinical Samples

The performance of the DENV-3 RT-LAMP assay was evaluated using 95 serum RNA samples previously characterized by triplex RT-qPCR. This panel comprised 65 DENV-3-positive samples (Ct < 32) and 30 samples negative for DENV, CHIKV, and ZIKV by RT-qPCR. Among the DENV-3-positive samples, the mean Ct value was 20.95 and the median Ct value was 20.58. All samples yielded positive amplification of the 18S rRNA internal control by RT-LAMP. Using the DENV-3 RT-LAMP assay, 59 of 65 RT-qPCR-positive samples were correctly identified as positive (TP), corresponding to a Ct range of 10.08–30.87. All 30 RT-qPCR-negative samples were correctly classified as negative by RT-LAMP (TN). Six RT-qPCR-positive samples were not detected by RT-LAMP (FN), with Ct values ranging from 22.95 to 30.34 (Table 2). These samples were retested in duplicate, and the results in both assays confirmed that six were false-negative. Representative RT-LAMP results for samples S16–S23, including the colorimetric detection and confirmation by agarose gel electrophoresis, are shown in Figure S4. The RT-LAMP DENV-3 system demonstrated a diagnostic sensitivity of 90.7% (95% CI: 80.98% to 96.54%) and a diagnostic specificity of 100% (95% CI: 88.43% to 100%).

### 3.4. Colorimetric Quantification of LAMP DENV-3

**Figure 4:**
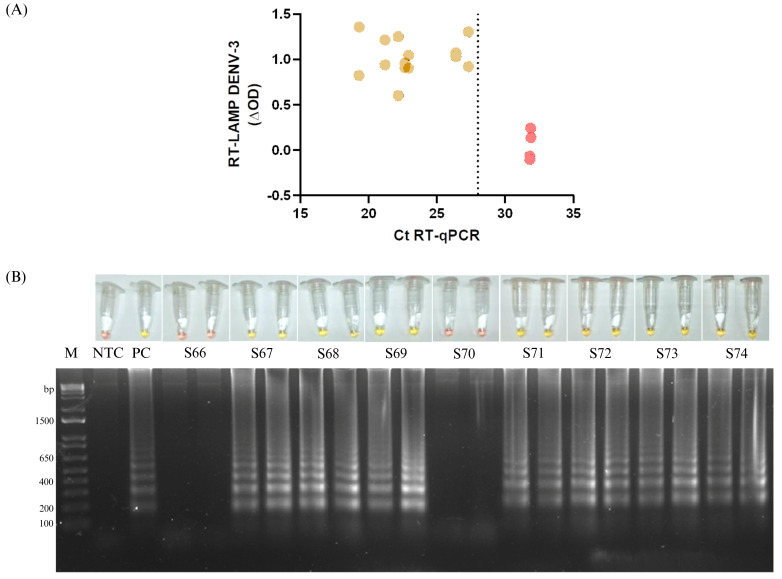
Quantitative colorimetric analysis of the DENV-3 RT-LAMP assay. (**A**) Scatter plot showing the relationship between the colorimetric signal (∆OD) of the DENV-3 RT-LAMP assay and the corresponding RT-qPCR Ct values obtained from nine freshly collected DENV-3-positive RNA samples. Yellow data points indicate positive RT-LAMP reactions, while red data points indicate negative reactions. The dotted vertical line denotes the estimated detection threshold of the RT-LAMP assay, corresponding to a Ct value of 28. (**B**) Representative colorimetric outcomes (upper panel) and gel electrophoresis of amplified products (lower panel) of the RT-LAMP reactions.

To exclude the possibility that assay sensitivity was affected by sample storage time, an approximate detection threshold was evaluated using nine fresh RNA of serum samples from the year 2025. These samples corresponded to the same RNA extracts that had already been used for diagnostic confirmation with the gold-standard assay. Colorimetric outputs of the DENV-3 RT-LAMP assay were quantitatively analyzed and compared with corresponding RT-qPCR Ct values. The optical density of the colorimetric RT-LAMP reactions was measured at 434 nm and 560 nm to quantify the pH-dependent redox state of phenol red. When ∆OD values were plotted against RT-qPCR Ct values, two clearly distinct clusters were observed, corresponding to the positive (n = 7) and negative (n = 2) results obtained by DENV-3 RT-LAMP assay (Figure 4). Positive samples clustered within ∆OD values ranging from 0.601 to 1.358 and exhibited Ct value < 30. In contrast, two false-negative samples showed ∆OD values between -0.066 and 0.138 and Ct value > 30.

## 4. Discussion

Over the past few decades, the reemergence of vector-borne diseases has been reported worldwide, driven by climate change as well as biotic, abiotic, and sociodemographic factors [19,20]. Among the endemic arboviruses in the Americas, DENV disease remains a major public-health concern [21]. Globally, dengue is often cited as one of the most consequential vector-borne diseases after malaria, with an estimated 3.9 billion people at risk of infection [4]. During 2023–2024, 14.6 million dengue cases were reported, reflecting a marked increase in incidence and highlighting intensified transmission, including increased circulation of DENV-3 in several settings [5]. In Mexico, 48,392 DENV-3 cases were confirmed in 2024 [22], consistent with one of the largest outbreaks reported in recent decades [21,23].

RT-qPCR is widely used as a reference method for arboviral genome detection in epidemiological surveillance and confirmatory diagnosis [24]. However, RT-qPCR requires specialized equipment, highly trained personnel, and laboratory infrastructure, which limits implementation in many point-of-care settings [25]. Therefore, accessible, sensitive, specific, and affordable molecular diagnostic methods are needed. LAMP has emerged as a promising alternative because it can be performed under isothermal conditions with simplified instrumentation and rapid readouts [11,12,26]. Here, we developed and evaluated a RT-LAMP assay for DENV-3 detection and assessed its performance as a potential screening tool to support clinical diagnosis in Southeastern Mexico.

To facilitate result interpretation and reduce assay cost, we implemented a colorimetric RT-LAMP format based on phenol red, a pH-sensitive indicator that enables visual detection of amplification without specialized optical equipment [27,28]. Colorimetric readouts are particularly advantageous for POC testing, as they allow rapid, instrument-free interpretation while maintaining assay robustness. Previous studies have described RT-LAMP assays for DENV detection using primer sets targeting the 3′ UTR and employing turbidity and/or fluorescence-based readouts [13,14,15]; this is a region reported to be highly conserved across DENV genomes [29]. In this study, primers were designed against a conserved segment of the DENV-3 3′ UTR, and analytical evaluation showed a preliminary limit of detection of 1 × 10^3^ copies/reaction, comparable to reported LAMP-based systems [30,31].

Because DENV, chikungunya virus (CHIKV), and Zika virus (ZIKV) co-circulate in Mexico [32], analytical specificity is critical; we evaluated LAMP cross-reactivity using DENV-1, DENV-2, DENV-4, ZIKV, and CHIKV-RNA and observed amplification only for DENV-3, supporting the high specificity of the assay within the panel tested. This is particularly relevant because differential diagnosis based on clinical presentation alone is limited by overlapping signs and symptoms among arboviral infections [32,33], underscoring the need for molecular tests that enable pathogen- and serotype-specific detection.

Current diagnostic algorithms often include a multiplex/triplex RT-qPCR assay to differentiate DENV, ZIKV, and CHIKV, followed by an additional assay to determine DENV serotype [34]. In this context, a DENV-3-specific RT-LAMP assay could reduce turnaround time and conserve laboratory resources by enabling rapid serotype-specific screening when DENV-3 circulation is suspected or increasing. Recent reports have associated increased DENV-3 circulation with higher numbers of dengue cases with warning signs and severe dengue in some settings [35,36,37], and improved serotype confirmation may support epidemiological studies examining associations between serotype, viral load, and clinical severity. Clinical validation was performed using retrospective serum RNA samples provided by the LAVE-UIMY, a regional reference laboratory for the diagnosis of arbovirus circulating in Southeastern Mexico (Quintana Roo, Yucatán, Campeche, and Tabasco). Using serum samples from this region highlights the value of coordinated work between diagnostic laboratories, hospitals, and biobanks for the development and evaluation of new diagnostic tools [38,39]. In our dataset, the assay showed a diagnostic sensitivity of 90.7% and a specificity of 100% relative to RT-qPCR. The observed sensitivity is consistent with ranges reported for LAMP-based diagnostic assays [40,41,42]. Regarding the analytical sensitivity, we selected 10^3^ copies/reaction as the preliminary limit of detection when synthetic recombinant plasmid was employed, as an orange, yellowish color was observed, therefore the LoD could be lower. Typically, an acute infection contains ≥ 10^5^ viral targets [43,44,45,46,47]. For this reason, this sensitivity is accepted for detecting actual samples from patients.

During evaluation with clinical samples, six RT-qPCR-positive samples tested negative by RT-LAMP. Because these samples were residual sera stored at LAVE/UIMY and to exclude the possibility that RNA degradation from storage time or freeze–thaw cycles contributed to reduced detection an approximate the clinical detection threshold was determined, we related colorimetric signal (ΔOD calculated from absorbance at 434 and 560 nm [18]) to RT-qPCR Ct values for clinical RNA samples. Positive RT-LAMP results clustered primarily at Ct values below ~30. Using CDC performance estimates for the triplex RT-qPCR kit (LoD corresponding to Ct ≈ 37.07 and 4.36 × 10^4^ genome copies) as a reference [48], our data suggest that RT-LAMP positivity in this setting corresponds roughly to samples with Ct ≤ 30 (approximately ≥ 1 × 10^6^ genome copies). Therefore, samples with Ct value > 30 may fall below the effective detection range of this colorimetric RT-LAMP format and may be more susceptible to false-negative results when RNA integrity is suboptimal. Importantly, the intended use case for RT-LAMP is rapid testing of fresh samples only. Epidemiological studies indicate that a substantial proportion of acute dengue cases present with medium-to-high viral loads (e.g., >10^5^–10^6^ genome copies) [43,44,45,46,47], suggesting that the RT-LAMP DENV-3 assay could detect a large fraction of infections during outbreaks, particularly early in the acute phase. This point-of-care detection method has a practical context. A calculated turnaround time considering hands-on time (RNA extraction, incubation and readout) is approximately 90 min. Also, it is important to consider only a lab bath maintaining constant temperature. Our group has implemented RT-LAMP, including the purification of RT and *Bst* enzymes [49], which has substantially reduced the cost compared to commercial kits.

The LAMP system with colorimetric product detection is a promising tool for rapid diagnosis and epidemiological surveillance of DENV [50]. However, its implementation depends on highly specialized primer design to target six to eight contiguous conserved regions [10]. Given the high mutation rate of DENV [51] and the dynamic epidemiological landscape, designing primers that detect all four serotypes with the same specificity and sensitivity continues to be challenging. Therefore, due to the high interserotype and intraserotype variability, primers have been designed in one of the most conserved genomic regions, the 3′UTR region [51]. Recent advances in computational primer design and predictive bioinformatic tools may help overcome key limitations of LAMP by improving primer selection, reducing assay development time, and enabling faster deployment of reliable assays in diverse epidemic settings [52,53].

Other limitations should be considered for implementation at the point of care. First, future work should evaluate simplified RNA preparation methods that do not require specialized extraction kits and/or assess direct testing protocols. Second, the RT-LAMP reagent mix is sensitive to freeze–thaw cycles; lyophilization could improve stability, simplify transport, and expand applicability in low-resource settings. Third, broader serotype coverage remains essential. Developing and validating complementary RT-LAMP assays for DENV-1, DENV-2, and DENV-4 would support a more comprehensive diagnostic panel suitable for settings where serotype dynamics shift over time. Ongoing work by our group is focused on expanding and prospectively evaluating these assays in primary care settings; however, to consider the implementation of this method for routine diagnostics, in terms of biosafety, it is still necessary to control contamination, so workflows should separate pre/post-amplification and include negative controls. Additionally, larger multicenter studies with standardized protocols are needed to evaluate the diagnostic performance, reproducibility and clinical utility of these DENV-LAMP assays in point-of-care settings.

## 5. Conclusions

The developed RT-LAMP DENV-3 assay demonstrated high specificity and good preliminary sensitivity when validated with clinical samples from Southeastern Mexico. Our RT-LAMP system could be effectively implemented in point-of-care settings to meet the need for rapid turnaround time, minimal equipment requirement and easy-to-interpret diagnostic systems. The RT-LAMP DENV-3 test could be used in prospective studies as a screening test and expanded to additional DENV serotypes to enhance its diagnostic capabilities and applicability.

## Figures and Tables

**Table 1:** Primers for DENV-3 RT-LAMP.

Target	Primer Name	Sequence 5′-3′
DENV-3	F3	GCCACATTAAGCCACAGTA
	B3	GTTGTGTCATGGGAGGG
	FIP	TGGCTTTTGGGCCTGACTTCTTTTTTGAAGAAGCTGTGCTGCCTG
	BIP	CTGTAGCTCCGTCGTGGGGATTTTCTAGTCTGCTACACCGTGC
	LF	CCTTGGACGGGGCT
	LB	GGAGGCTGCAAACTGTG

F3/B3: External primers; FIP/BIP: internal primers; FL/BL: loop primers.

**Table 2:** Contingency table of RT-LAMP DENV-3 assay.

DENV-3 RT-LAMP	RT-qPCR-Positive Samples	RT-qPCR-Negative Samples	Total
Positive samples	59 (TP)	0 (FP)	59
Negative samples	6 (FN)	30 (TN)	36
Total	65	30	95

True positives (TP); True Negatives (TN); False Positives (FP) and False Negatives (FN).

## Data Availability

The raw data supporting the conclusions of this article will be made available by the authors on request.

## Supplementary Materials

The following supporting information can be downloaded at: https://www.mdpi.com/article/10.3390/pathogens15040359/s1, Figure S1. RT-LAMP primer design targeting the DENV-3 3′ UTR. Schematic representation of the hybridization regions of the RT-LAMP primer set designed for detection of DENV-3. Internal primers (FIP and BIP) consist of the F2 and B2 sequences, respectively, linked at their 5′ ends to the complementary sequences F1c and B1c through a poly-T linker. F3/B3: external primers; LF/LB: loop primers. Arrows indicate the direction of DNA synthesis for eachprimer. Figure S2. Neighbor-joining tree with 426 sequences of 218 pb of 3′UTR RT-LAMP DENV-3 blank region. Sequences available in NCBI Genebank from American isolates, DENV-3 reference sequence (NC001475.2). Figure S3. Logo sequence of the amplification region of the DENV-3 RT-LAMP system. Figure S4. DENV-3-positive samples by RT-LAMP. RNA serum samples from patients diagnosed as positive or negative for DENV-3 by RT-qPCR were used to validate the RT-LAMP DENV-3. A representative gel showing the positive and negative results of samples S16 to S23 is shown. M (marker); NTC (negative control); PC (positive control).

## Abbreviations

The following abbreviations are used in this manuscript:

DENV-3	Dengue virus serotype 3
RT-LAMP	Reverse Transcription Isothermal Amplification
Bst	*Bacillus stearothermophilus*
IMSS	Instituto Mexicano del Seguro Social
LAVE	Epidemiological Surveillance Support Laboratory
rRNA	Ribosomal RNA
TP	True positives
TN	True Negatives
FP	False Positives
