# Supplementary material for: Detection of Dengue Virus Serotype 3 Using a Colorimetric Reverse Transcription Loop-Mediated Isothermal Amplification Assay: Evaluation with Clinical Samples from Southeastern Mexico
Source: Pathogens. 2026 Mar 28;15(4):359. doi: 10.3390/pathogens15040359 (PMC13118920; doi:10.3390/pathogens15040359)
Supplement: Supplementary file 1 [file pathogens-15-00359-s001.zip › pathogens-4161849-supplementary.pdf]

Supplementary Figures

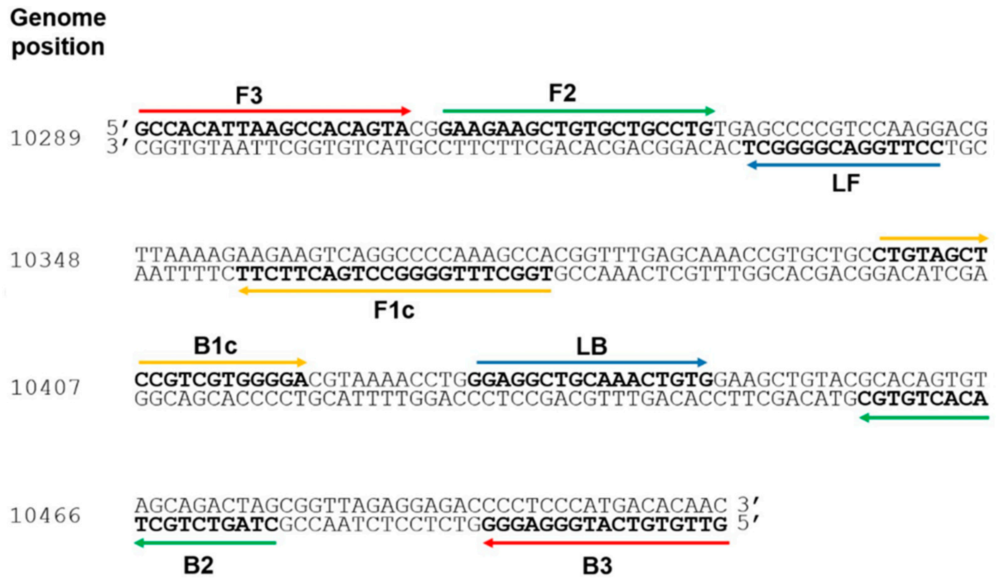

Figure S1. RT-LAMP primer design targeting the DENV-3 3′ UTR. Schematic representation of the hybridization regions of the RT-LAMP primer set designed for detection of DENV-3. Internal primers (FIP and BIP) consist of the F2 and B2 sequences, respectively, linked at their 5′ ends to the complementary sequences F1c and B1c through a poly-T linker. F3/B3: external primers; LF/LB: loop primers. Arrows indicate the direction of DNA synthesis for each primer.

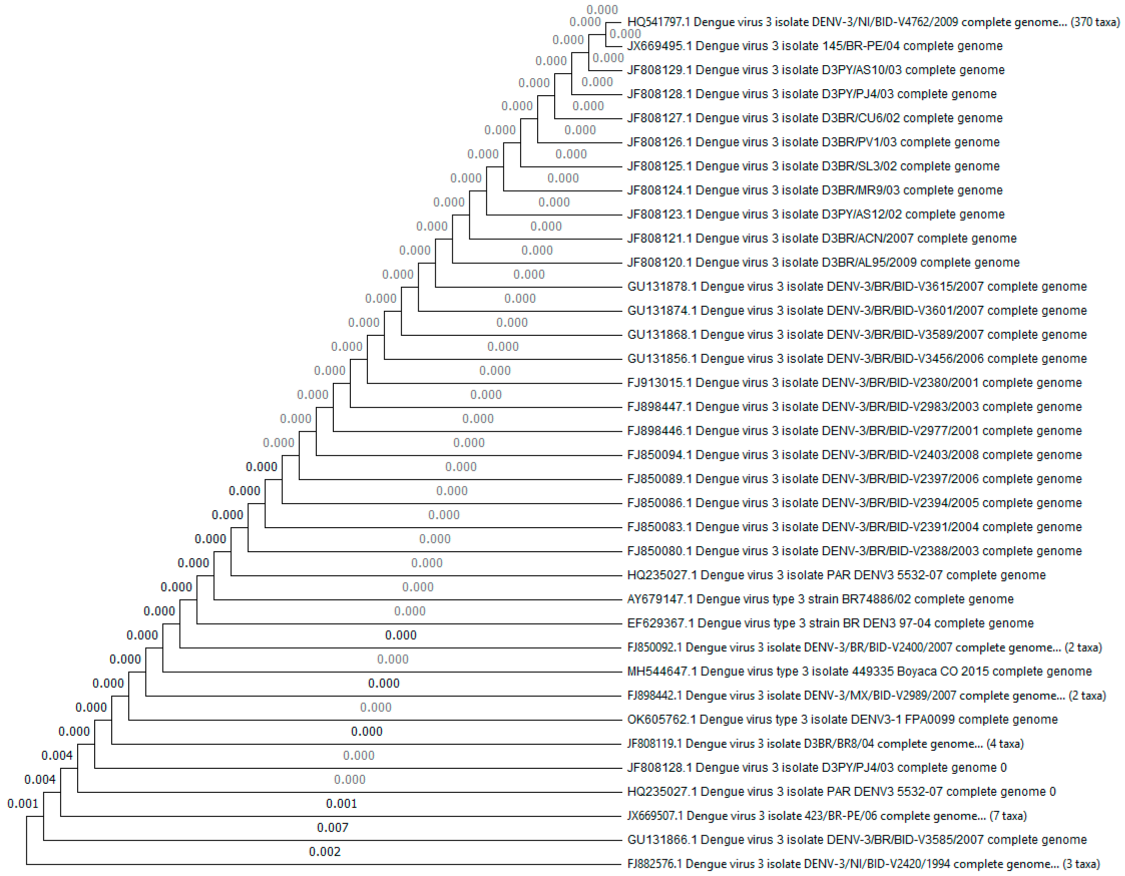

Figure S2. Neighbor-joining tree with 426 sequences of 218 pb of 3′UTR RT-LAMP DENV-3 blank region. Sequences available in NCBI Genebank from American isolates.

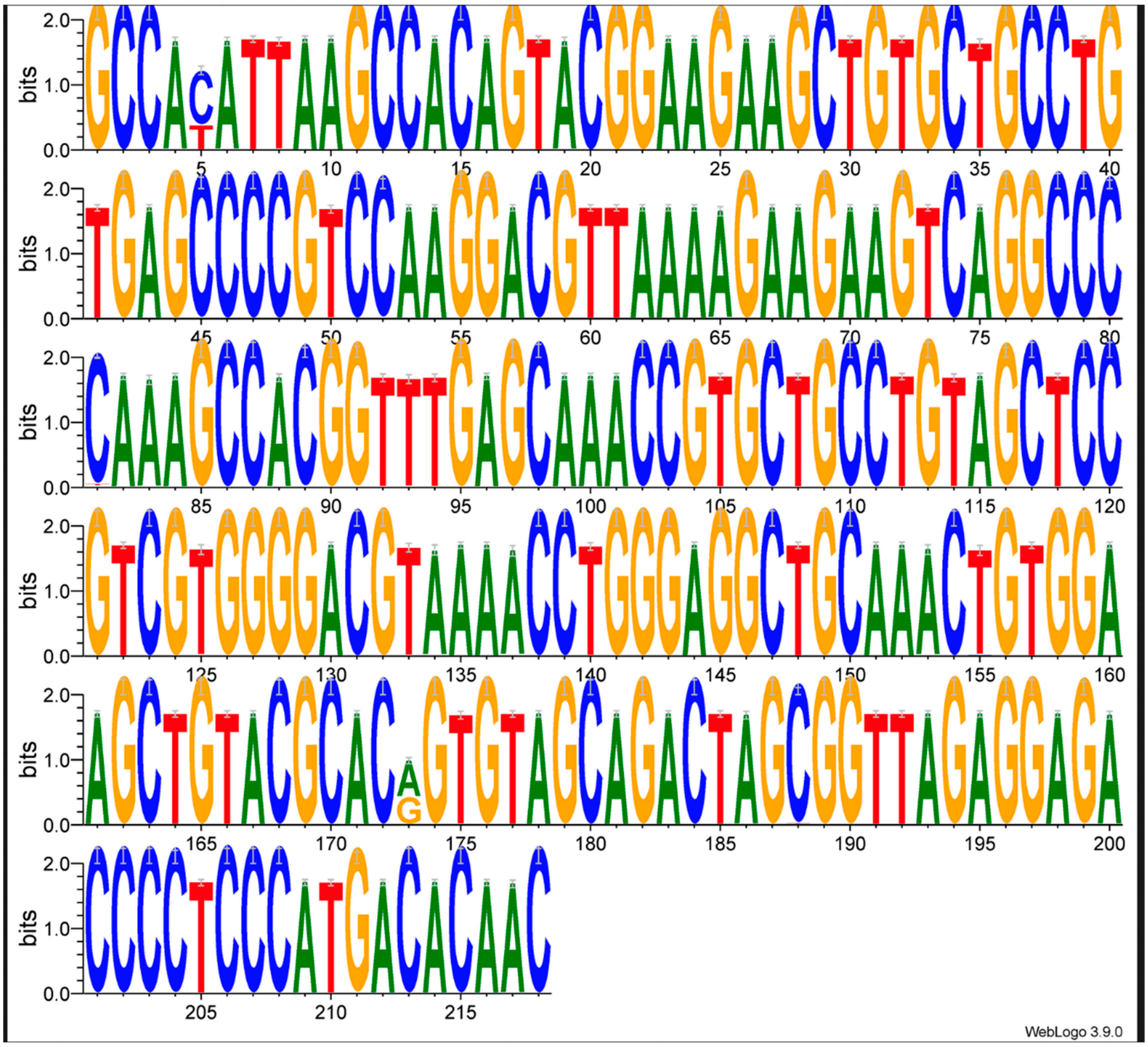

Figure S3. Logo sequence of the amplification region of the DENV-3 RT-LAMP system.

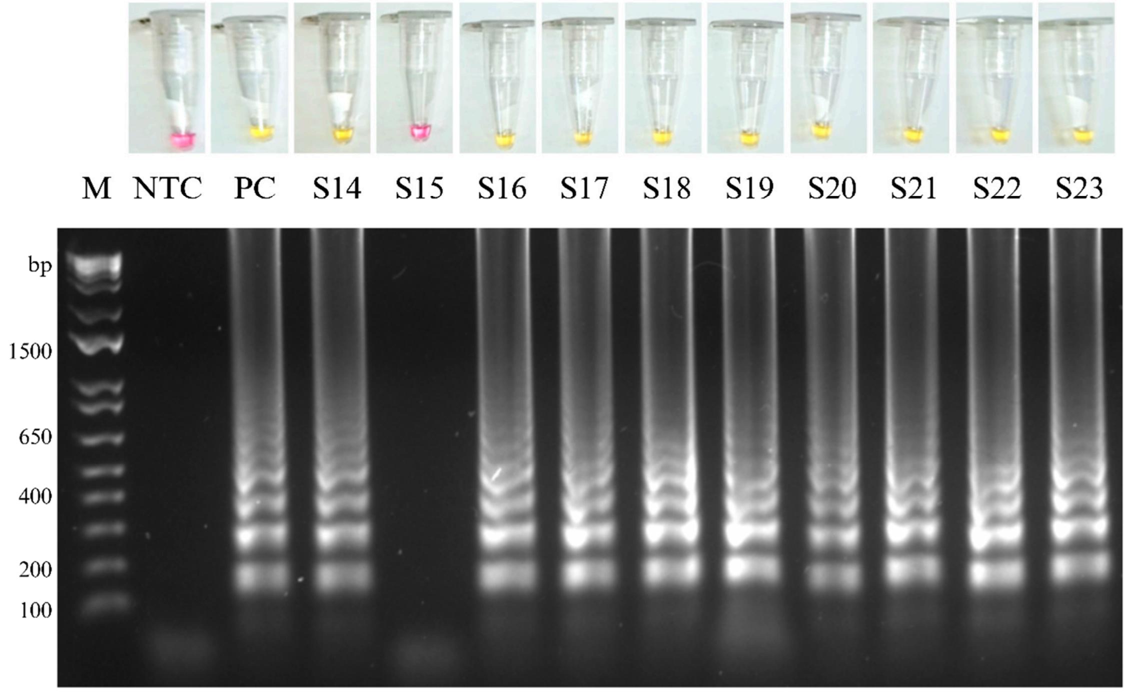

Figure S4. DENV-3 positive samples by RT-LAMP. RNA serum samples from patients diagnosed as positive or negative for DENV-3 by RT-qPCR were used to validate the RT-LAMP DENV-3. A representative gel showing the positive and negative results of samples S14 to S23 is shown. Yellow reactions are considered positive and red reactions are considered negative. M (marker); NTC (negative control); PC (positive control).
